# Luteolin supports osteogenic differentiation of human periodontal ligament cells

**DOI:** 10.1186/s12903-019-0926-y

**Published:** 2019-10-26

**Authors:** He Quan, Xiaopeng Dai, Meiyan Liu, Chuanjun Wu, Dan Wang

**Affiliations:** Economic & Technological Development Area Clinic, Yantai Stomatological Hospital, No. 11 Songshan Road, Yantai, 264000 Shandong Province China

**Keywords:** Periodontal ligament, Cell differentiation, Wnt signaling pathway, Osteogenesis, Luteolin

## Abstract

**Background:**

Previous research revealed that luteolin could improve the activation of alkaline phosphatase (ALP) and osteocalcin in mouse osteoblasts. We aimed to determine the effect of luteolin on osteogenic differentiation of periodontal ligament cells (PDLCs).

**Methods:**

Cultured human PDLCs (HPDLCs) were treated by luteolin at 0.01, 0.1, 1, 10, 100 μmol/L, Wnt/β-catenin pathway inhibitor (XAV939, 5 μmol/L) alone or in combination with 1 μmol/L luteolin. Immunohistochemical staining was performed to ensure cells source. Cell activity and the ability of osteogenic differentiation in HPDLCs were determined by MTT, ALP and Alizarin Red S staining. Real-time Quantitative PCR Detecting System (qPCR) and Western blot were performed to measure the expressions of osteogenic differentiation-related genes such as bone morphogenetic protein 2 (BMP2), osteocalcin (OCN), runt-related transcription factor 2 (RUNX2), Osterix (OSX) and Wnt/β-catenin pathway proteins members cyclin D1 and β-catenin.

**Results:**

Luteolin at concentrations of 0.01, 0.1, 1, 10, 100 μmol/L promoted cell viability, ALP activity and increased calcified nodules content in HPDLCs. The expressions of BMP2, OCN, OSX, RUNX2, β-catenin and cyclin D1 were increased by luteolin at concentrations of 0.01, 0.1, 1 μmol/L, noticeably, 1 μmol/L luteolin produced the strongest effects. In addition, XAV939 inhibited the expressions of calcification and osteogenic differentiation-related genes in HPDLCs, and 1 μmol/L luteolin availably decreased the inhibitory effect.

**Conclusion:**

1 μmol/L luteolin accelerated osteogenic differentiation of HPDLCs via activating the Wnt/β-catenin pathway, which could be clinically applied to treat periodontal disease.

## Introduction

As a common oral disease, periodontal disease is a main cause to tooth loss and could lead to local or even systemic effects [1]. Periodontal disease mainly promotes the regeneration of periodontal tissue, producing a certain number of healthy periodontal ligament cells (PDLCs) functioning as the primary basis for the repair of periodontal disease [2]. Derived from the mesoderm, PDLCs are the most abundant cells in the periodontal membrane and also the main cell source for the attachment between gingiva and root surface after periodontal treatment [3]. Additionally, PDLCs can not only promote the formation of new main fibers and cementum, but also play a vital role in the reconstruction of alveolar bone [4]. The osteogenic differentiation of PDLCs is also essential in the regeneration of periodontal tissues [5]. Among the conduction pathways, Wnt/β-catenin signaling pathway, which plays a significant role in embryonic development, organ formation, tumor formation and bone reconstruction [6], could activate the expression of downstream target gene cyclin D1 in the nucleus, promotes the activity of osteoblasts and the mineralization of extracellular matrix by regulating directional differentiation of osteoblasts and the expressions of specific genes [7].

It has been reported that luteolin, which often exists as glycosylation in nature, could affect osteogenic differentiation [8]. Previous studies also showed that luteolin was a natural tetrahydroxyl flavonoid compound with a molecular weight of 286.23 kD [9]. luteolin was initially isolated from the leaves, stems and branches of resedaceae, however, researchers found that luteolin could also be extracted from a variety of natural medicinal materials, vegetables and fruits such as honeysuckle, wormwood, celery and cabbage [10]. In pharmacology, luteolin is a multifunctional complex that has a positive medicinal effect, for example, anti-cancer, anti-inflammatory, regulating immunity function, resisting oxidation and reducing osteoclast activity [11].

In treating periodontal disease, as auxiliary measures to oral mechanical treatment, pharmaceutical drugs can improve therapeutic efficacy. However, some western medicines such as antibiotics and other commonly used drugs have certain toxic and side effects, thus, as antibiotic resistance becomes stronger, their effectiveness in treating periodontal disease is limited to some extent [12]. Studies have proved that various traditional Chinese medicine herbs had specific therapeutic effects on treating periodontal diseases such as radix scutellariae [13] and cinnamaldehyde [14].

As luteolin could protect human bronchial epithelial cells via activating nuclear factor erythroid 2-related factor 2 (Nrf2) pathway, some scholars believed that luteolin can be used as a medicine for the prevention and treatment of lung cancer [15]. Nash et al. [16] pointed out that the luteolin extracted from tea could increase the mineral content in human osteoblasts. In addition, according to the study of Abbasi et al. [17], a low concentration of luteolin could protected osteoblasts from oxidative stress induced by high glucose. In dental field, Liu L et al. [18] found that luteolin could effectively maintain the pluripotency of PDLC by activating related pathways. Though, studies on the application of luteolin in osteoblastic cells increased gradually, the effect of luteolin on osteoblastic differentiation of PDLCs has not yet been investigated. Therefore, this study mainly explored the effect of different concentrations of luteolin on human PDLCs (HPDLCs), and analyzed its effects on osteogenic differentiation and Wnt/β-catenin signaling pathway. Our findings provide a new understanding on the treatment of periodontal diseases.

## Methods

This study was approved by the Ethics Committee of Yantai Stomatological Hospital, and all donors signed the informed consent.

### Cell culture

HPDLCs were obtained from healthy human third molars, the teeth came from six donors aged between 18 and 35 years old. All patients had their teeth removed due to orthodontic requirement, and they did not have concomitant dental, pulp or periodontal diseases. The teeth were washed 3 times by sterile phosphate buffer saline (PBS), and then the periodontal ligament was separated from the middle third of the root surface using a blade in an aseptic ultra-clean table, and the PDL was cut into thin slices of 1 mm^3^. The PDL tissue was cultured in dulbecco’s modified eagle medium (DMEM, Gibco, Carlsbad, USA) containing 10% fetal bovine serum (FBS, Millipore, USA), 100 mg/mL streptomycin and 100 U/mL penicillin (Gibco, USA) at 37 °C in a humid environment with 5% CO_2_. HPDLCs passage was performed by digestion with 0.05% ethylene diamine tetraacetic acid (EDTA) plus 0.25% trypsin (Sigma, USA). The medium was changed every 3 days until the cells were separated from the tissues and filled 80% of the well plate. Cells used in each experiment came from only one donor from his third to sixth generations.

### Cell identification

To determine the source and characteristic of HPDLCs, immunofluorescence detection was performed on the HPDLCs at 3rd passage. Preliminary experimental analysis showed that 5000 cells per well plate in a 24-well plate were optimal for cell identification. Briefly, 1.5 mL DMEM medium containing 10% FBS was added into the cells, which were washed twice by PBS buffer, fixed with 4% paraformaldehyde for 30 min at room temperature and blocked by human mesenchymal stem cell characterization kit (Millipore, Billerica, MA, USA). Next, vimentin and cell keratin (mouse, vimentin BM0135, cytokeratin BM0030, 1:200, BosterBio, Wuhan, China) were added into the cells and held overnight at 4 °C. After being rinsed in PBS buffer for 3 times, the cells were incubated with fluorescein isothiocyanate and secondary antibody horseradish peroxidase-conjugated goat anti-mouse IgG (A0216, 1:500, Beyotime, Suzhou, China) at room temperature for 45 min [19]. Then, the cells were redyed with 4, 6-diamino-2-phenylindole (DAPi, 1:100, Vector Laboratories, Burlingame, CA, USA). Fluorescence microscopy (BX-41, Olympus Optical, Tokyo, Japan) was used for image analysis.

### Treatment of cells

HPDLCs at a density of 2 × l0^5^/mL were inoculated on three 96-well plates [20] in an incubator at 37 °C with 5% CO_2_ until 70~80% confluence was reached. Each group in this study had six multiple holes and the surrounding holes were filled with sterile PBS solution. Luteolin (batch number: 111520–200,201, purity> 99%, China Food and Drug Administration, Beijing, China) was dissolved by dimethyl sulfoxide (DMSO, Sigma, USA), and the different concentrations (0.01, 0.1, 1, 10, 100 μmol/L) of luteolin [21, 22], Wnt/β-catenin pathway inhibitor XAV939 (5 μmol/L, purity> 98%, Sigma, USA) [23], 1 μmol/L of luteolin in combination with XAV939 (luteolin was first incubated for 20 min and added with XAV939) were added into well plates and served as positive groups. In addition, untreated cells in control group were incubated with PBS buffer and considered as a negative control, compared with other groups in this study. The treated cells used in the following experiments were confirmed by preliminary experiments, as well as the time frame had the best experimental effect at the corresponding treatment time.

### Cell viability analysis

3-(4),-5-dimethylthiazole-2-acyl)-2, 5-diphenyltetrazole ammonium bromide (MTT) assay was used to determine the viability of HPDLCs. After 24, 48 and 72 h of treatment, 10 μL MTT (1 mg/mL, Sigma, USA) was added to the cells and held for 4 h in the dark at 37 °C. Then, the formazan crystals dissolved in 200 mL DMSO were added to each well and held for 10 min. Finally, the optical density (OD) of each well was measured at 490 nm wavelength by enzyme-linked immunoassay (ELX808, BioTek, Vermont, USA), and the average value was calculated.

### Alkaline phosphatase (ALP) activity analysis

Alkaline phosphatase (ALP) activity analysis was conducted to determine the osteogenic differentiation ability of HPDLCs. Cells were cultured in differentiation medium (Sigma, USA) containing DMEM medium (10% FBS), 10^− 7^ mol/L dexamethasone, 50 μg/mL ascorbic acid Vc and 10 mmol/L sodium β-glycerophosphate for 72 h at 37 °C with 5% CO_2._ After 72 h of incubation, the cells were rinsed 3 times with PBS buffer and fixed with 4% polyformaldehyde at 4 °C for 10 min. Next, BCIP/NBT alkaline phosphatase coloring kit (Beyotime, Suzhou, China) was used for ALP staining according to the manufacturer’s instructions. After being incubated with luteolin for 3 d, the ALP activity of HPDLCs was measured using ALP kit (Jiancheng Bioengineering, Nanjing, China) and enzyme-linked immunoassay (ELX808, BioTek) at 520 nm wavelength.

### Mineralization characteristics analysis

Alizarin Red S staining was used to determine content of calcified nodules in HPDLCs. The HPDLCs treated for 24 h were cultured in a 35 mm Petri dish containing differentiation medium, which was changed every 2 days during 5 weeks. When mineralized nodules were formed, the cells were fixed with 4% polyformaldehyde for 30 min, stained by 0.1% Alizarin Red S (Sigma, USA) at pH 4.3 for 30 min at room temperature and rinsed with deionized water. The staining results were observed under a microscope using a digital camera (Nikon, Japan). Cetylpyridine chloride (CPC) method was applied to detect the content of calcium deposition, and the absorbance was measured using a multifunctional microplate reader (M1000 Pro, TECAN, Switzerland) at 560 nm.

### Quantitative polymerase chain reaction (qPCR)

qPCR assay was performed to detect the expressions of osteogenic differentiation-related genes in HPDLCs. After 72 h of treatment, Trizol reagent (Invitrogen, Carlsbad, California, USA) was used to extract the total RNA from the cells, and the purity and concentration of RNA were determined by spectrophotometer (Nano Drop Technologies ND-1000, Wilmington, Delaware, USA). Total RNA (1 μg) was extracted and synthesized into cDNA by performing reverse transcription at 37 °C for 15 min using PrimeScript™ RT reagent kit (Takara, Japan). SYBR PremixEx Taq Kit (TaKaRa, Japan) was used to carry out qPCR assays, and the reaction conditions were set as follows: pre-denaturation at 94 °C for 30 s, denaturation at 94 °C for 10 s, annealing at 60 °C for 30 s, extension at 72 °C for 3 min and final extension at 72 °C for 10 min. The primer base sequences (Gene Pharma, Shanghai, China) used were bone morphogenetic protein 2 (BMP2), osteocalcin (OCN), runt-related transcription factor 2 (RUNX2), Osterix (OSX), cyclin D1 and β-catenin and listed in Table 1. Each reaction was carried out for three times using glyceraldehyde-3-phosphate dehydrogenase (GAPDH) as an internal control, and the data were analyzed by the 2^-ΔΔCT^ method [24].

**Table 1:** Primer base sequence

Gene	Forward (5′-3′)	Reverse (5′-3′)
BMP2	TATTTGGATAAGAACCAGACATTG	GAAAGAAGAACAACAAACCATCA
OCN	AGCAAAGGTGCAGCCTTTGT	GCGCCTGGGTCTCTTCACT
RUNX2	GAGATTTGTGGGCCGGAGTG	CCTAAATCACTGAGGCGGTC
OSX	ACCTACCCATCTGACTTTGCTC	CCACTATTTCCCACTGCCTTG
Cyclin D1	TGATGCTGGGCACTTCATCTG	TCCAATCATCCCGAATGAGAGTC
β-catenin	AAAATGGCAGTGCGTTTAG	TTTGAAGGCAGTCTGTCGTA
GAPDH	AACGGATTTGGTCGTATTGG	TGGAAGATGGTGATGGGATT

### Western blotting (WB) analysi***s***

Western blotting (WB) analysis was performed to detect proteins related to osteogenic differentiation and Wnt/β-catenin pathway. After 3 days of treatment, all proteins were extracted from cells on ice by RIPA lysis buffer (Beyotime, Suzhou, China) containing 1 mmol/L phenylmethanesulfonyl fluoride (PMSF) and centrifuged for 20 min (10,000 *g*) at 4 °C. Protein content was determined by bicinchoninic acid (BCA) protein assay kit (Fude, China). A total of 30 μg protein lysates were separated by 8% sodium dodecyl sulfate polyacrylamide gel electropheresis (SDS-PAGE, Beyotime, China) and transferred to polyvinylidene fluoride (PVDF, Beyotime, China) membrane, which was blocked for 2 h at room temperature in 3% skimmed milk. Primary antibodies (BMP2 (ab14933, 1:2000, abcam, USA; https://www.abcam.cn/bmp2-antibody-ab14933.html), OCN (ab13420, 1:1000, abcam, USA; https://www.abcam.cn/osteocalcin-antibody-ocg3-ab13420.html), RUNX2 (ab76956, 1:1000, abcam, USA; https://www.abcam.cn/runx2-antibody-ab76956.html), Osterix (OSX, ab22552, 1:2000, abcam, USA; https://www.abcam.cn/sp7-osterix-antibody-chip-grade-ab22552.html), cyclin D1 (ab134175, 1:2000, abcam, USA; https://www.abcam.cn/cyclin-d1-antibody-epr2241-c-terminal-ab134175.html), β-catenin (ab8226, 1:2000, abcam, USA; https://www.abcam.cn/beta-actin-antibody-mabcam-8226-loading-control-ab8226.html) and GAPDH (ab8245, 1:2000, abcam, USA; https://www.abcam.cn/gapdh-antibody-6c5-loading-contr ol-ab8245.html)) were added to the membrane and held overnight at 4 °C. After washing the cells with TBST for three times, goat anti-mouse IgG antibody labeled with horseradish peroxidase (A0216, 1:1000; Beyotime, Suzhou, China; https://www.beyotime.com/product/A0216.htm) and goat anti-rabbit IgG H&L (HRP) (ab205718, 1:2000, abcam, USA; https://www.abcam.cn/goat-rabbit-igg-hl-hrp-ab205718.html) were added and held for 2 h at room temperature. ECL chemiluminescence kit (Millipore, USA) was used for the exposure of the membrane, and Bio-Rad ChemiDoc XRS + Imaging System was used to analyse the signal intensity of gel band.

### Statistical analysis

SPSS 20.0 software was used for statistical analysis. Metrological data were expressed by mean ± standard deviation (SD). Before t-test, Shapiro-Wilk method was used to analyze whether the data in this study conform to normal distribution and whether the data were in line with the normal distribution. The difference between groups was analyzed by t-test or one-way ANOVA with the LSD test. The difference was defined as statistically significant when *P* < 0.05.

## Results

### Characteristics of HPDLCs and effect of luteolin on cell activity

**Fig. 1:**
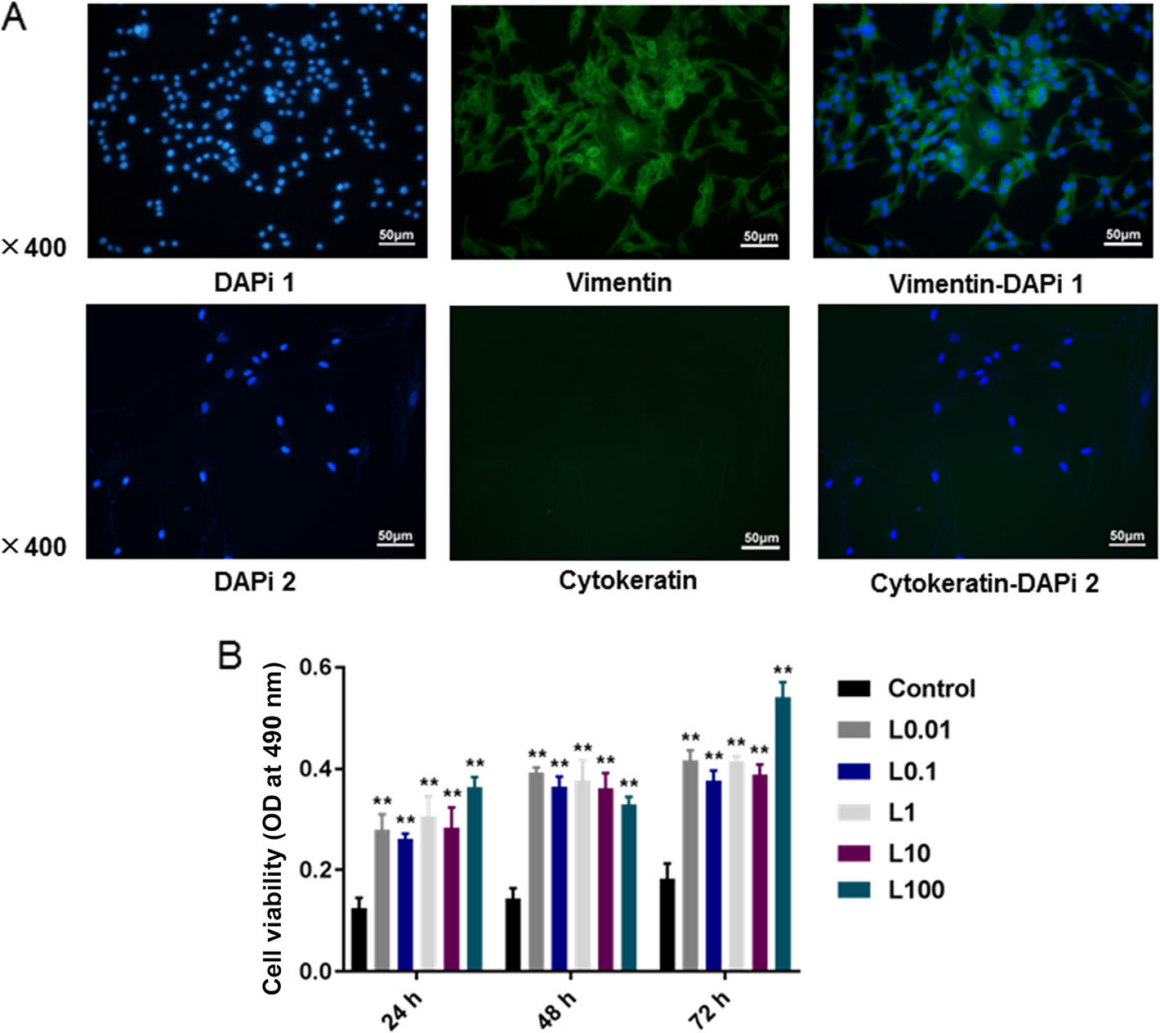
**a** Result of immunohistochemical staining in HPDLCs (× 400): vimentin staining was positive but keratin staining was negative. **b** MTT assay was performed to detect the effects of luteolin (L) at different concentrations (0, 0.01, 0.1, 1, 10, 100 μmol/L) on the viability of HPDLCs after 24, 48, and 72 h of treatment. ^**^P < 0.001, vs. Control

The results of immunofluorescence detection showed that vimentin staining was positive, while keratin staining was negative in HPDLCs (Fig. 1a). After 24, 48, 72 h of luteolin treatment, the results of MTT assay showed that luteolin at different concentrations (0.01, 0.1, 1, 10, 100 μmol/L) significantly increased the viability of HPDLCs (*P* < 0.001), and no obvious difference was identified in the function of luteolin at different concentrations on HPDLCs proliferation at the same time point (Fig. 1b).

### Effect of luteolin on osteogenic differentiation of HPDLCs

**Fig. 2:**
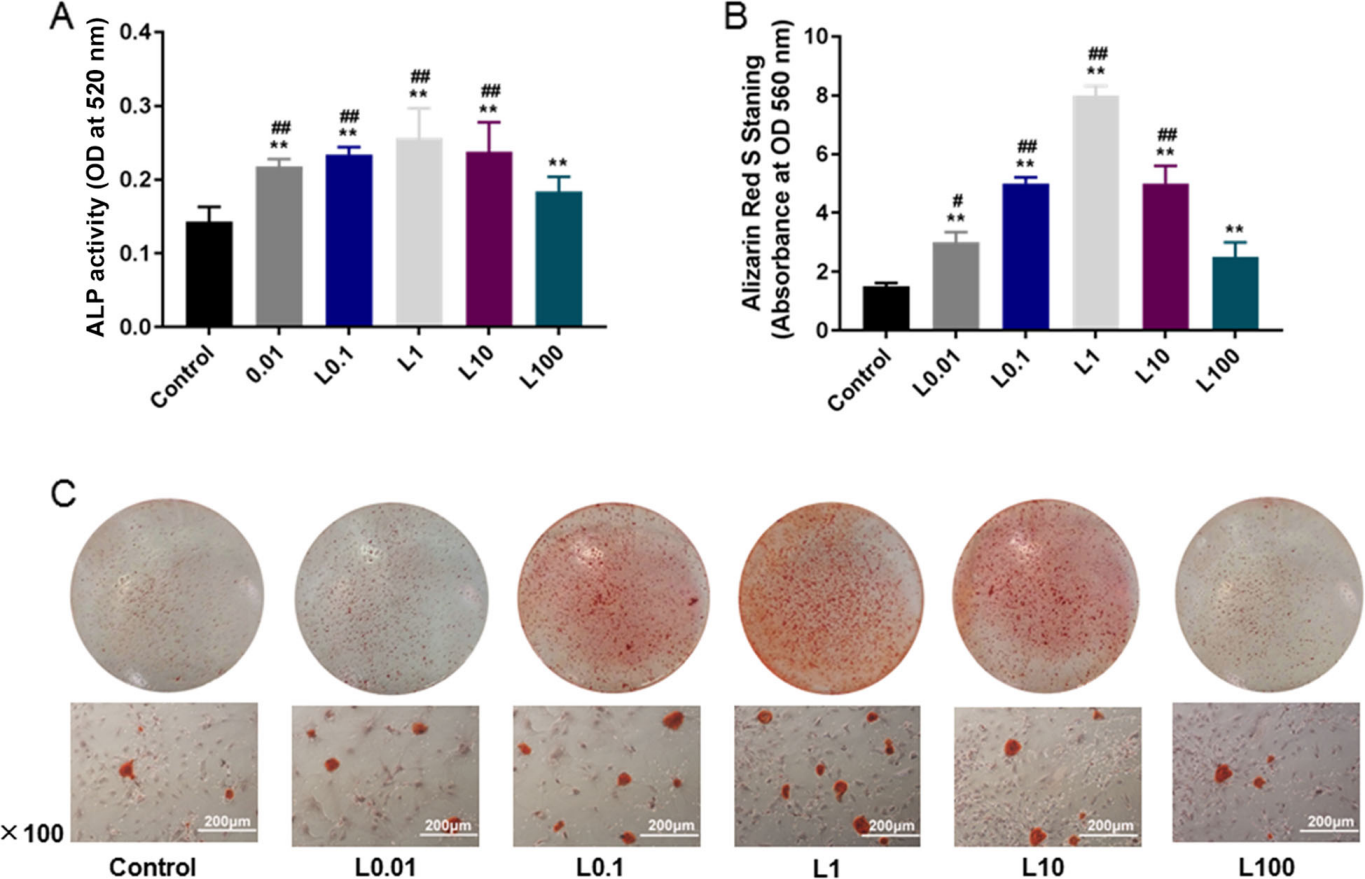
Effect of luteolin (L) on osteogenic differentiation in HPDLCs. The effect of luteolin at different concentrations (0, 0.01, 0.1, 1, 10, 100 μmol/L) on osteogenic differentiation in HPDLCs were studied by ALP activity (**a**), calcified nodules formation and calcium deposition (**b**, **c**) according to the ALP and Alizarin Red S staining assay.^**^P < 0.001, vs. Control. ^##^*P* < 0.001, ^#^*P* < 0.05, vs. L100

The data in our study showed that different concentrations (0.01, 0.1, 1, 10, 100 μmol/L) of luteolin significantly enhanced ALP activity (*P* < 0.001) and increased the content of calcified nodules in HPDLCs (*P* < 0.001). Moreover, low concentration (0.01, 0.1, 1, 10 μmol/L) produced a stronger effect than high concentration at 100 μmol/L (*P* < 0.05) and luteolin at 1 μmol/L had the strongest effect (Fig. 2a, b and c).

### Effects of luteolin on the expressions of genes related to osteogenic differentiation and Wnt/β-catenin pathway protein of HPDLCs

**Fig. 3:**
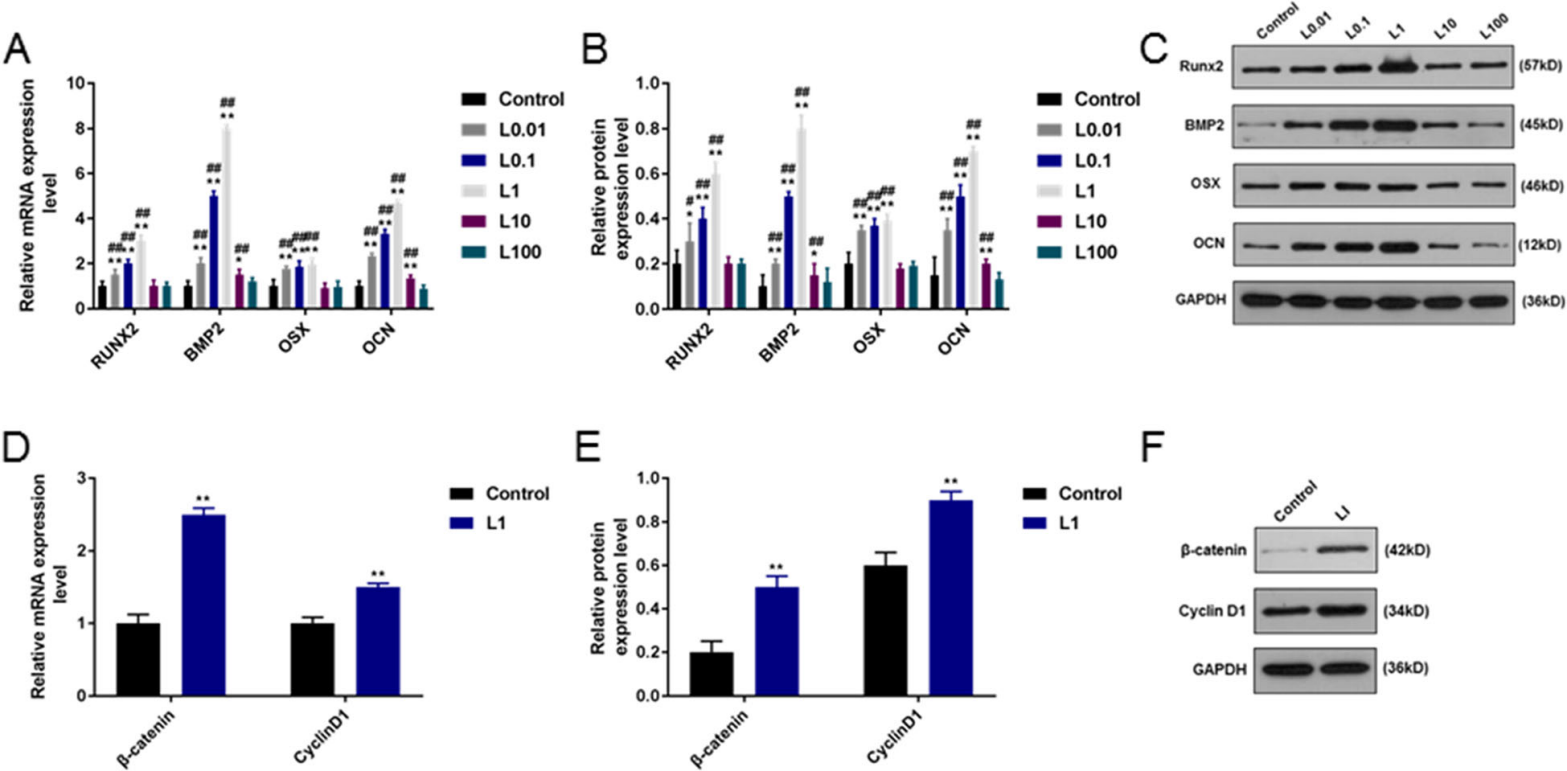
Effect of luteolin (L) on the expressions of genes related to osteogenic differentiation and Wnt/β-catenin pathway protein of HPDLCs. **a**, **b**, **c** qPCR and WB analyses of osteogenic differentiation-related genes (BMP2, OCN, RUNX2, OSX) and **d**, **e**, **f** the expressions of Wnt/β-catenin pathway proteins’ (cyclin D1, β-catenin) in HPDLCs with the treatment of luteolin at different concentrations (0, 0.01, 0.1, 1, 10, 100 μmol/L). ^**^P < 0.001, ^*^P < 0.05, vs. Control. ^##^*P* < 0.001, ^#^*P* < 0.05, vs. L100

Results of qPCR and WB analysis indicated that the relative mRNA and protein expressions of BMP2, OSX and OCN were significantly increased by luteolin at concentrations of 0.01, 0.1, 1 and 10 μmol/L (*P* < 0.001), and the expression of RUNX2 was greatly increased by luteolin at concentrations of 0.01, 0.1, and 1 μmol/L (*P* < 0.05). However, high concentration of luteolin (100 μmol/L) had no significant effect on the relative mRNA and protein expressions of genes related to osteogenic differentiation (Fig. 3a, b and c). Thus, 1 μmol/L luteolin could produce the optimal effect on the osteoblastic differentiation. Furthermore, the relative mRNA and protein expressions of β-catenin and cyclin D1 were significantly increased by 1 μmol/L luteolin (*P* < 0.001, Fig. 3d, e and f).

### Effect of Wnt/β-catenin pathway inhibitor XAV939 on HPDLCs

**Fig. 4:**
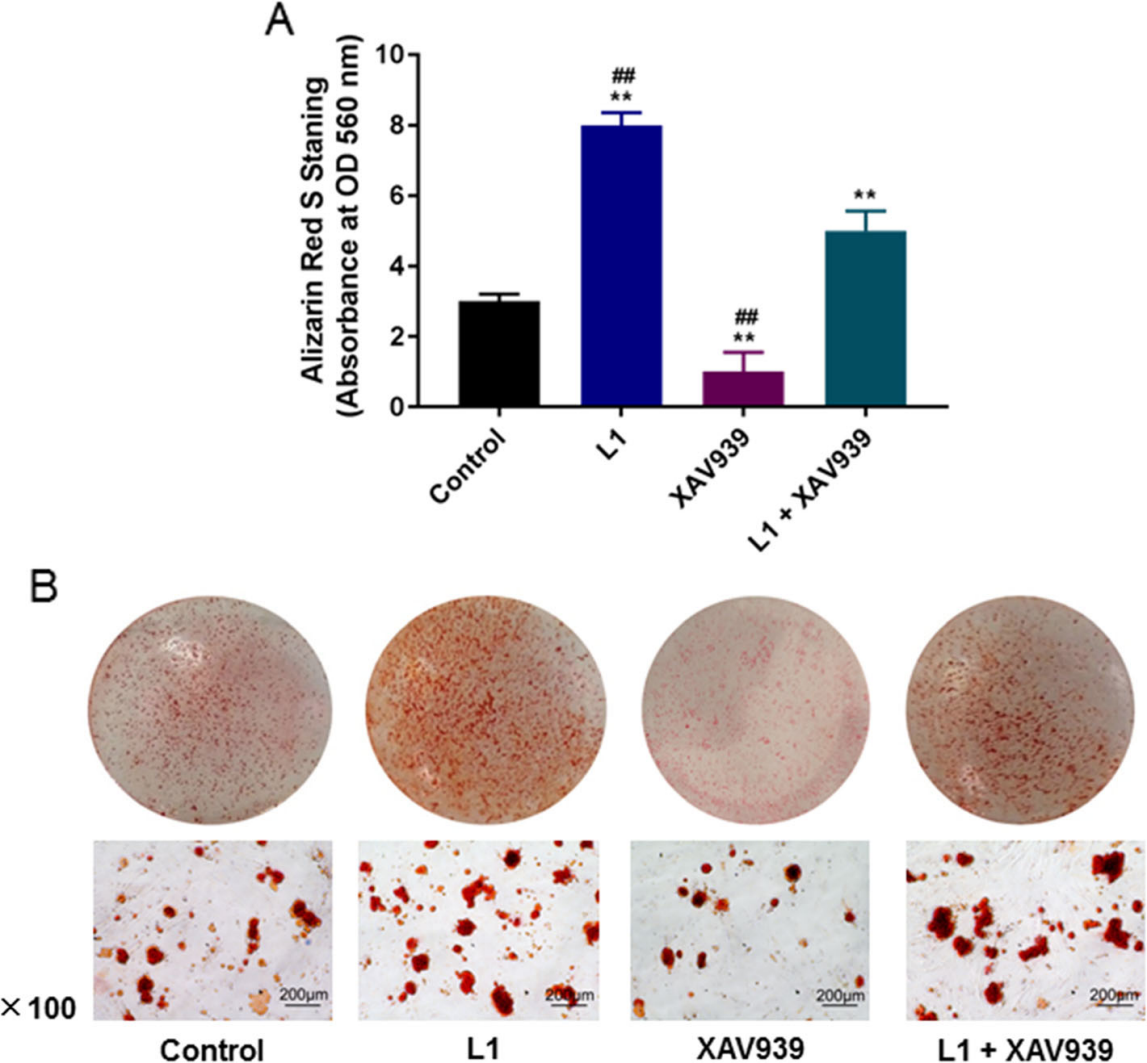
Effect of Wnt/β-catenin pathway inhibitor XAV939 on HPDLCs (L). **a**, **b** Alizarin Red S staining was used to detect the formation of calcified nodules and calcium deposition in HPDLCs after treatment of 1 μmol/L luteolin, 5 μmol/L XAV939 or the combination of the two. ^**^P < 0.001, vs. Control. ^##^*P* < 0.001, vs. L1 + XAV939

From the data of Alizarin Red S staining assay, 1 μmol/L luteolin promoted the calcification of HPDLCs (*P* < 0.001), while XAV939 inhibited the calcification of cells (*P* < 0.001). Thus, luteolin could effectively limit the inhibiting action of XAV939 (*P* < 0.001, Fig. 4a and b).

### Effect of Wnt/β-catenin pathway inhibitor XAV939 on the expression of gene related to osteogenic differentiation

**Fig. 5:**
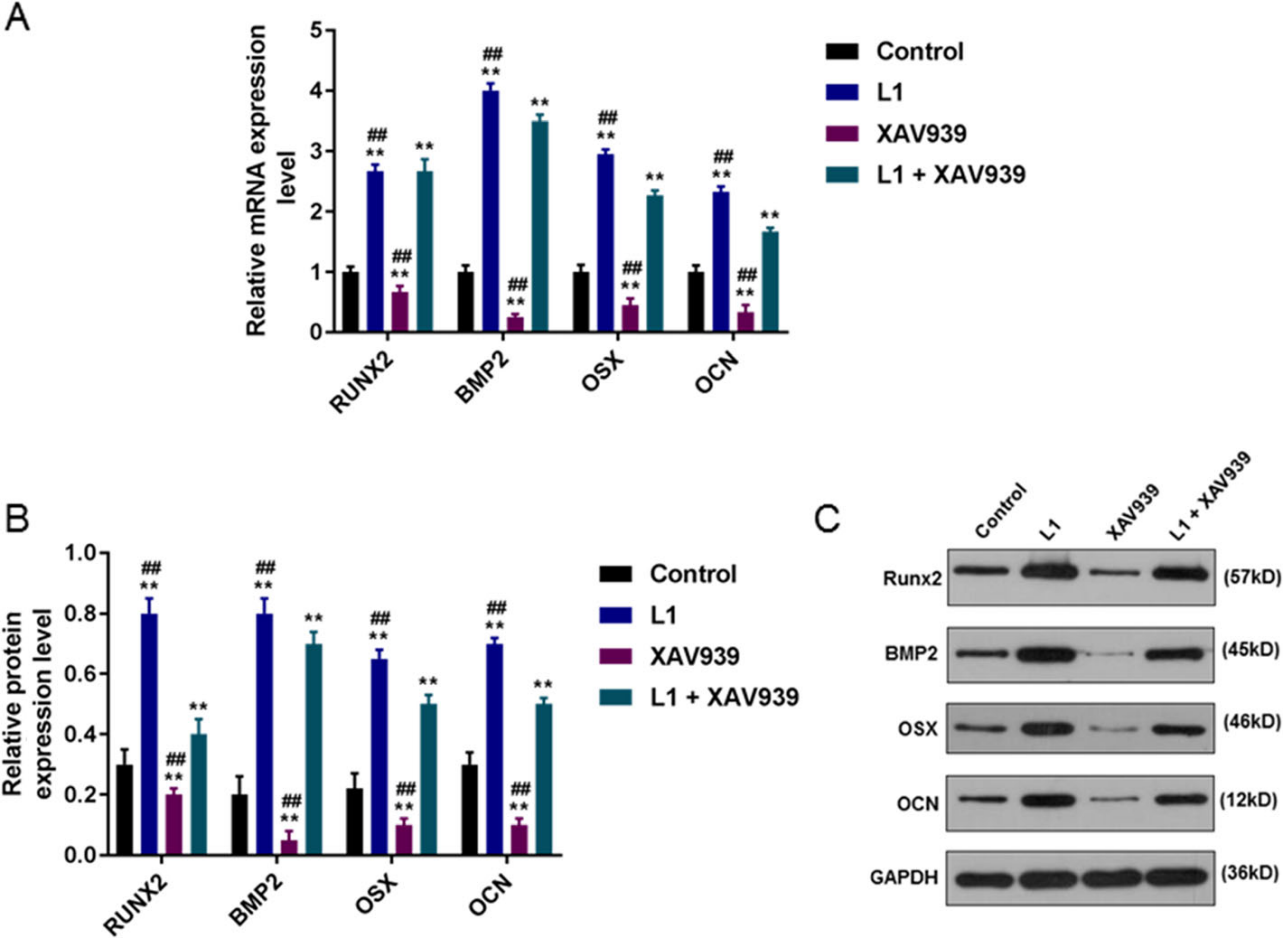
Effect of Wnt/β-catenin pathway inhibitor XAV939 on the expressions of genes related to osteogenic differentiation. **a**, **b**, **c** The expressions of BMP2, OCN, RUNX2 and OSX in HPDLCs after treatment of 1 μmol/L luteolin (L), 5 μmol/L XAV939 or combination of the two were detected through qPCR and WB assays. ^**^P < 0.001, vs. Control. ^##^*P* < 0.001, vs. L1 + XAV939

The results of qPCR and WB assay showed that 1 μmol/L luteolin significantly increased the relative mRNA and protein expressions of osteogenic differentiation-related genes (BMP2, OCN, RUNX2 and OSX) (*P* < 0.001), while 5 μmol/L XAV939 greatly decreased the relative mRNA and protein expressions of osteogenic differentiation-related genes (*P* < 0.001). Moreover, 1 μmol/L luteolin effectively reduced the inhibitory effect of XAV939 (*P* < 0.001, Fig. 5a, b and c).

## Discussion

HPDLCs, which are abundant in periodontal tissues, have multi-directional differentiation potential such as generating fibroblasts and osteoblasts and cementum cells [25]. Some studies confirmed that bone nodules could be formed from HPDLCs under certain condition, and that bone-related proteins such as ALP, bone sialoprotein, osteocalcin could also be expressed [26, 27]. Furthermore, previous studies proved that periodontal tissue regeneration and repair was mainly dependent on the number and osteogenic differentiation ability of HPDLCs in periodontal tissue [28]. According Sun et al. [29], luteolin could alleviate the cytotoxicity induced by methylacetaldehyde, thus protecting MC3T3-E1 osteoblasts cells. Based on these studies, we speculated that luteolin had an effect of promoting osteogenic differentiation of HPDLCs. In addition, we also determined the optimal concentration of the drug and the related mechanism.

In this study, we investigated the characteristics of HPDLCs by immunofluorescence detection, the results revealed that vimentin staining was positive but keratin staining was negative, and our result was in line with a previous study [30], proving that the cells came from mesenchyme and the cell source was reliable. Then, luteolin at different concentrations were added to the HPDLCs and co-cultured, and the propagation capacity of the cells was detected by MTT assay. We found that luteolin at different concentrations could promote the generation of HPDLCs. However, the proliferation effect of luteolin on HPDLCs did not concern drug concentration, which might be explained by the significant difference in drug concentration gradient. In the following study, the concentration gradient can be reduced to the optimal medicine concentration.

ALP, which is a non-specific phosphomonoesterase, is generally present in human body, and it is a critical biomarker reflecting the osteogenic activity of cells. On the other hand, ALP, which can be used to examine the osteogenic differentiation function of HPDLCs, also plays an important role in cell mineralization [31], similarly, using Alizarin Red S staining is also a method for the detection of mineralization, especially in the measurement of late osteoblast differentiation impact [32]. Study by Lei et al. [33] demonstrated that the proliferation, osteogenic differentiation and mineralization of osteoblasts could be enhanced under some conditions. The results in our investigation revealed that luteolin at different concentrations could promote ALP activity and calcify nodules formation in HPDLCs, and that the effect of luteolin at low concentrations (0.01, 0.1, 1, 10 μmol/L) was stronger, suggesting that a certain dose of luteolin could enhance the effect of mineralization and osteoblast differentiation in the cells. In animal models, Kim et al. [34] treated ovariectomized mice with luteolin, and found that luteolin could obviously increase the density and content of bone mineral in the femur of mice and reduce osteoclast differentiation. HPDLCs may comprise adult stem cells or subsets and have the effect of promoting osteogenic differentiation into osteoblasts under the effect of luteolin, resulting in a higher ALP activity and the development of a quantitative content of mineralized nodules.

Furthermore, qPCR and WB experiments were carried out to determine the expressions of genes. BMP2 is capable of inducing the differentiation of undifferentiated mesenchymal stem cells into chondroblasts and osteoblasts [35]. OCN is related to maturation of osteoblasts [36]. RUNX2 plays a significant role in the early proliferation of osteoblasts [37], and OSX is seated downstream of RUNX2 and also plays a key role in late osteoblastic differentiation and maturation [38]. Jia LI et al. [39] showed that luteolin could not only promote the osteogenic differentiation and proliferation of MC3T3-E1 cells, but also up-regulate the mRNA expression levels of RUNX2 and OCN. In this study, we demonstrated that luteolin at 0.01, 0.1, 1 μmol/L increased the correlative mRNA and protein expressions of BMP2, OCN, RUNX2 and OSX, and 1 μmol/L luteolin showed a relatively high performnace. Interestingly, 100 μmol/L of luteolin had no significant effects on the expressions of all osteogenic indicators, however, 10 μmol/L of luteolin increased the expressions of BMP2, OCN and OSX but did not have significant effect on the expression of RUNX2. Similarly, Liu L et al. [40] also found that luteolin affected the expressions of multipotent markers in dental pulp cells in a concentration-dependent manner. This proved that luteolin at a certain concentration could activate osteogenic differentiation of HPDLCs.

As for the Wnt/β-catenin signaling pathway, Rongrong et al. [41] discovered that Wnt/β-catenin signal pathway could activate the expression of BMP2 in osteoblasts. This study indicated that luteolin at 1 μmol/L had the strongest effect on promoting osteogenic differentiation and it could obviously stimulate the production of β-catenin and cyclin D1, suggesting that luteolin might promote HPDLCs differentiation into osteoblasts via activating the Wnt/β-catenin pathway. To further test our speculation, we used Wnt/β-catenin pathway inhibitor (XAV939) to cultivate HPDLCs, and the result showed that XAV939 decreased the amount of calcified nodules and the expressions of genes related to osteogenic differentiation. Moreover, luteolin was found to relieve the inhibitory action of XAV939. According to the study data of Tian et al. [42], XAV939 decreased the death of neuroblastoma cell lines via controling the Wnt/β-catenin pathway by blocking the signal pathway. Fujita et al. [43] also pointed out that XAV939 promoted the differentiation and maturation of osteoblasts in mice, which was reflected in the accessorial expressions of osteoblast-related genes. These results verified that the inhibition of Wnt/β-catenin could limit the differentiation of cells into osteoblasts.

## Conclusions

In conclusion, luteolin at certain concentrations could promote the proliferation and osteogenic differentiation of HPDLCs, increase the expressions of genes related to osteogenic differentiation and activate the Wnt/β-catenin pathway, noticeably, 1 μmol/L of luteolin had the strongest effect. Therefore, we recommended that luteolin of 1 μmol/L could be served as an optimal concentration to accelerate osteogenic differentiation of HPDLCs via activating the Wnt/β-catenin pathway. Thus, our results contribute to the clinical application of periodontal disease.

## Data Availability

The analyzed data sets generated during the study are available from the corresponding author on reasonable request.

## Abbreviations

ALP: Alkaline phosphatase
BCA: Bicinchoninic acid
BMP2: Bone morphogenetic protein 2
CPC: Cetylpyridine chloride
DMEM: Dulbecco’s modified eagle medium
EDTA: Ethylene diamine tetraacetic acid
FBS: Fetal bovine serum
HPDLCs: Human PDLCs
Nrf2: Nuclear factor erythroid 2-related factor 2
OCN: Osteocalcin
OD: Optical density
OSX: Osterix
PBS: Phosphate buffer saline
PDLCs: Periodontal ligament cells
PMSF: Phenylmethanesulfonyl fluoride
PVDF: Polyvinylidene fluoride
qPCR: Quantitative polymerase chain reaction
RUNX2: Runt-related transcription factor 2
SD: Standard deviation
SDS-PAGE: Sodium dodecyl sulfate polyacrylamide gel electropheresis
TBST: Tris buffered saline tween
